# Ocular Surface Ion-Channels Are Closely Related to Dry Eye: Key Research Focus on Innovative Drugs for Dry Eye

**DOI:** 10.3389/fmed.2022.830853

**Published:** 2022-03-03

**Authors:** Shuo Yang, Yaying Wu, ChunYang Wang, Xiuming Jin

**Affiliations:** Eye Center, Second Affiliated Hospital, School of Medicine, Zhejiang University, Hangzhou, China

**Keywords:** ocular surface, dry eye, ion channels, compounds, targeted therapy

## Abstract

Abundant ion-channels, including various perceptual receptors, chloride channels, purinergic receptor channels, and water channels that exist on the ocular surface, play an important role in the pathogenesis of dry eye. Channel-targeting activators or inhibitor compounds, which have shown positive effects in *in vivo* and *in vitro* experiments, have become the focus of the dry eye drug research and development, and individual compounds have been applied in clinical experimental treatment. This review summarized various types of ion-channels on the ocular surface related to dry eye, their basic functions, and spatial distribution, and discussed basic and clinical research results of various channel receptor regulatory compounds. Therefore, further elucidating the relationship between ion-channels and dry eye will warrant research of dry eye targeted drug therapy.

## Introduction

Ion channels are crucial for sensing temperature and mechanical and chemical stimuli and are important structures for transmitting information between cells (1–3). Dry eye (DE) is a multifactorial ocular surface syndrome that mainly manifests as a series of sensory abnormalities on the ocular surface, including pain, burning sensation, and increased sensitivity to foreign bodies (4, 5). Abnormal physiological functions of the corneal epithelium and nerve endings also contribute to DE pathogenesis (5–10). These abnormalities, along with the dysfunctional expression and function of ion channels on the surface of the eye, further aggravate subjective symptoms of discomfort from DE (11, 12).

Many ion channels exist in the cornea, conjunctival epithelial cells, and corneal nerve fibers (4, 11). Their main physiological function is to maintain the internal and external ecological balance of the cells, sense chemical, temperature, and pressure stimuli, and transmit information (11). At present, many studies have revealed the key role of ion channels present in ocular surface cells in the pathogenesis of DE, and some ion channel activators or inhibitors have shown promise in the targeted treatment of DE (11–17). This review focused on the research status and progress of studying ion channels related to the ocular surface and DE and summarized the importance of ion channels in the DE to provide insights on DE-targeted drugs.

## Distribution of De-Related Ion-Channels on the Ocular Surface

**Figure 1:**
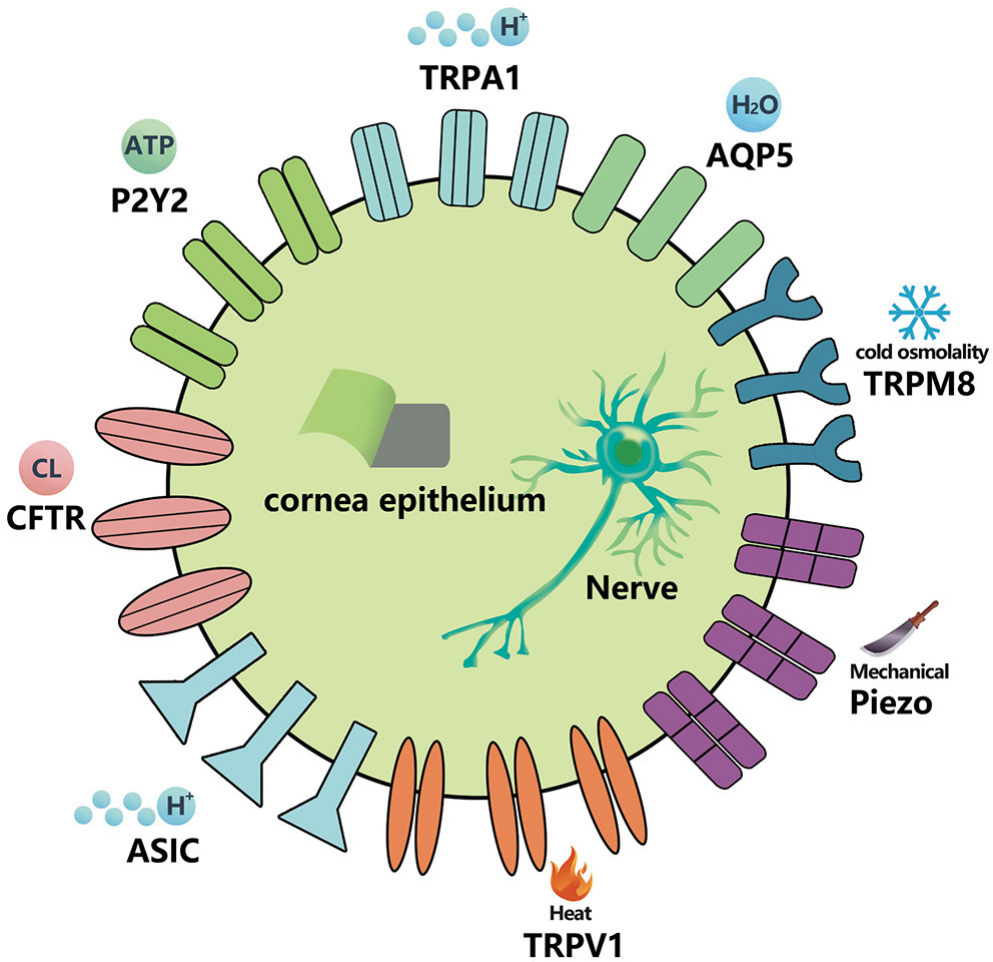
Ion-channels that exist in the cornea, conjunctival epithelial cells, and corneal nerve fibers. The specific stimuli activating each receptor channel class is shown (11, 18–20).

The stability of the ocular surface environment is an important factor in the pathological mechanisms of DE (5–9). The physiological state of the cornea, conjunctiva, and corneal nerve plays a considerable role in the occurrence and development of the DE, and the information transmission of related physiological functions depends on various ion channels at the cellular level (Figure 1) (11).

The trigeminal ganglion (TG) is distributed widely on the ocular surface through the eye meridian, especially in the cornea. There are multimodal nociceptors (~70%), mechanical nociceptors (15–20%), and temperature receptors (10–15%) at nerve endings, which can sense mechanical, thermal, and chemical stimuli within or near a harmful threshold (4, 11). At present, many studies have reported that the expression of ion channels is influenced by DE in corneal nerve endings, mainly including transient receptor potential (TRP) channels (TRPV1, TRPA1, and TRPM8), acid-sensing ion channels (ASICs), and mechanically gated ion channels (piezo2) (11–17).

Many ion channel receptor proteins also appear in corneal epithelial cells. At present, the ion channels related to DE include TRPV1, TRPM8, TRPA1, cystic fibrosis transmembrane conductance regulator (CFTR), chloride channels, among others (11, 13–16). In addition, receptor proteins closely related to the physiological functions of ion channels, such as purine and purinergic receptors [G protein coupled adenosine receptors (ARS), P2Y receptors (p2yrs), and ATP gated P2X receiver ion channels (p2xrs)], show potential in DE-targeted therapy (17). Aquaporins (AQPs), proteins involved in the balance and stability of the water inside and outside the cells, also exist in the corneal epithelium. Recently, it has been found that they are closely related to the pathogenesis of DE (21).

Goblet cells in the conjunctival epithelium secrete mucin (MUC5AC) and some non-mucin proteins, including peroxidase, trefoil factor, and defensin. As an important component of tear film formation, mucin maintains the conjunctival and corneal epithelial microenvironment (22–25). Studies have shown that purinergic gated ion channel proteins are expressed in the conjunctival epithelium and goblet cells and participate in the inflammatory response of NLRP3 (26). Mergler et al. detected human conjunctival cells and found that conjunctiva expressed TRPV1, TRPV2, and TRPV4 channels, implying novel drug targets for DE therapeutics (27).

## Polymodal Nociceptors and DE

Polymodal nociceptors are widely present in cornea and nerve fibers. They can be activated by external stimuli, such as harmful mechanical factors, heat, and chemical stimuli. Simultaneously, they are also sensitive to endogenous chemical mediators and inflammatory cells (11). In DEs, polymodal nociceptors can sense changes of temperature, inflammatory stimulation, and osmotic pressure caused by DEs, and regulate the wetting of the ocular surface. According to these characteristics, the screening of TRP family receptor block or activation-related compounds is of great significance for the development of DE-related ocular surface-targeted drugs. It has been reported that these receptors are closely related to DE, as described in the following paragraphs.

### Transient Receptor Potential A1 (TRPA1)

TRPA1, also known as ANKTM1, is an ion channel receptor of TRP that participates in nociceptive temperature and mechanical perception (28–30). TRPA1 activation causes calcium influx and plays a role in persistent and allergic inflammation (30, 31). Katagiri et al. found that TRPA1 mechanisms are involved in the sensitivity of ocular responsive trigeminal brain neurons in the model for tear defect DE (32).

### TRP Cation Channel Subfamily V Member 1 (TRPV1)

TRPV1 participates in polymodal nociceptors' sensory conduction (33). Previous studies have confirmed that TRPV1 is an ion channel related to sensations including pain and pruritus, and diseases like asthma (34–38). As a multi-sensory receptor, TRPV1 is activated by capsaicin, acidity, heat damage, and a hypertonic external environment (33). Eye irritation is a common clinical symptom of DE, and the TRPA1 inhibitor has shown great potential in easing peripheral nerve pain (39). Masuoka et al. have reported that chronic lacrimal deficiency sensitizes the response mediated by TRPV1 in corneal epithelial cells, which may be related to hyperalgesia caused by noxious stimulation in water-deficient DEs (40). In the DE model, overexpression of TRPV1 in TRPM8+ sensory neurons leads to cold hyperalgesia in corneal and non-corneal tissues without affecting their thermal sensitivity and promotes the release of neuropeptide P to signal cold response nociception (41). The increase in tear permeability and ocular surface inflammatory reactions are important mechanisms for the pathogenesis of DE (4–9). In addition, hyperosmotic stress (HOS) induces EGFR, MAPK, and NF signaling pathways through the TRPV1 channel and further mediates the increase of the pro-inflammatory cytokine interleukin (IL)-6 and chemokine IL-8 (42). Hua et al. found that the TRPV1 inhibitor capsaicin significantly reduced inflammatory tumor necrosis factor (TNF)-α, IL-1β, and IL-6 stimulated by human corneal endothelial cells (HCECs) (43). They further reported osmo-protectors to suppress inflammatory responses via the TRPV1 pathway in HCECs exposed to HOS (43). The targeted drug regulation of TRPV1 activity or its signal medium may be utilized as a novel method to inhibit the inflammatory response of DE syndrome. The mRNA expression levels of TRPA1, TRPV1, ASIC1, and ASIC3 were upregulated in the TG ocular branches of rigorously treated DE mice. After the intervention of the DE disease (DED) mouse model with the TRPV1 blocker (capsazepine), a reduction in the multimodal response of the cornea to heat, cold, and acid stimulation, and a further reduction in eye pain-associated anxious states were observed (44). Another study found that 1.125% syl1001, a novel short interfering RNA targeting TRPV1, can greatly improve ocular surface disease index scores and is tolerated well (45). Bereiter et al. clarified that TRPV1 plays an important role in mediating the enhanced nociceptive behavior in DE, which is beneficial for treating ocular surface stimulation pain in patients with moderate to severe DEs (46). These data support the effectiveness of blocking TRPV1 in treating tear deficiency and relieving chronic eye pain in DED, and is a potential choice for DED-targeted therapy.

### ASIC3

ASICs are hydrogen ion–gated cation channels that are easily activated and opened by extracellular H^+^ ions, instigate Na^+^ ion influx, lead to cell depolarization and excitation, and participate in the sensitization process of moderate acid stimulation (47–51). Previous ASIC-related studies have mostly focused on the protection of the optic nerve and retinal injury. Inhibition of ASICs can reduce retinal ischemia-reperfusion and optic nerve injury (52, 53). In DE research, the possible role of the ASIC channel was recognized during a recent study. ASIC3 was shown to activate several corneal multimode sensory nerve fibers, significantly increase the blink and tear rate, and mediate acid stimulation and inflammatory pain on the ocular surface (54). ASICs are present in corneal sensory neurons. An acidic pH depolarizes these neurons to stimulate the action potential. ASIC blockers eliminate nociceptive behaviors caused by moderate acid stimulation (54). Even in the allergic keratoconjunctivitis model, ASIC3 blocking can also significantly reduce nociceptive behavior and reduce nociceptor sensitization during inflammation (54).

## Mechano-Nociceptors and DE

In the corneal nerve fiber axons, 20–30% of the receptors are mechanical nociceptors sensitive to mechanical pressure and activated by pressure perception (11, 55). Piezo channel protein, a member of the mechanically gated cation channel family, is the main baroreceptor on the ocular surface (56). It can be divided into two subtypes: piezo1/fam38a and piezo2/fam38b. Bron et al. found that piezo2 expression occurred in ~26% of TG neurons and 30% of corneal afferent neurons (55). Morozumi et al. confirmed that piezo1 and especially piezo2 are common in the corneal epithelium and optic nerves (57). Piezo inhibitors can protect against optic nerve damage caused by high intraocular pressure (57). Corneas of patients with DEs are sensitive to temperature changes and mechanical stimulation. As a common clinical symptom of DE, corneal pain may be related to the influence of chronic inflammation on the corneal pain conduction pathway. The piezo2 channel existing in the corneal nerve is directly involved in alleviating acute corneal mechanical injury, and local regulation of piezo2 helps ease the related pain caused by ocular surface mechanical stimulation (58).

## Cold Receptors and DE

Cold receptors account for 10.15% of corneal neurons. They are sensitive to ocular surface temperature, continuously produce action potentials, and are regulated by dynamic changes in temperature (11). TRPM8 is a member of the cation channel family of TRP. As a cold sensory receptor, TRPM8 mainly senses temperature changes on the ocular surface and participates in temperature sensing, thermoregulation, and sensing pain caused by cold (11, 14, 16, 59). TRPM8 is also involved in the pathophysiology of DE and has anti-hypersensitivity and antipruritic effects (15, 60). On the ocular surface, evaporation of tears and changes in temperature lead to the activation of TRPM8, which further regulates basic tear secretion (61).

A study has found that in corneal refractive surgery and moderate DE, cold-heat receptors seem to mainly be affected, which can cause the major unpleasant feeling of DE (39). The corneal cold-sensitive neurons were closely related to the function of the TRPM8 channel in the injury response. The enhanced functional expression of the TRPM8 channel in primary sensory neurons of the trigeminal nerve could lead to an increase in tear rate and DE sensation (62). The dry conditions changed the sensitivity of neurons to menthol (its activator), resulting in desensitization to a cold-induced response, which could lead to reduced tear production that is harmful to patients with DEs (63). Moreover, certain interactions exist between TRP pathways. Khajavi et al. showed that the activation of TRPM8 can reduce TRPV1 activity, which may play a therapeutic role in the treatment of TRPV1-mediated inflammatory hyperalgesia, colitis, and DE syndrome (64). Arcas et al. reported the direct agonistic effect of tacrolimus on TRPM8 activity, which explains the anti-inflammatory effect of TRPM8 channels on the ocular surface of DEs (65). In related clinical studies, it was communicated that TRPM8 activation had a positive effect on DEs. A TRPM8 receptor agonist called cryosim-3 (C3, 1-diisopropylphosphorylnonane) can significantly increase tear secretion and improve DE symptoms (66). Yoon et al. also confirmed that C3 can significantly improve the symptoms of DE associated with menstrual pain, which is ineffective in routine treatments (67). In addition, a warm compress containing menthol can continuously increase the amount of tears and tear film stability in patients with DEs (68).

However, it seems paradoxical that patients with DED are more sensitive to cold compared to patients with non-DED. The duration of DED, rather than age, is an important factor in sensitivity to cold. Corneal cold receptor sensitivity decreased with an increase in DED duration (69). Fakih et al. found that severe DED mice had cold hyperalgesia, which was consistent with the high expression of TRPM8 mRNA in the TG. Chronic m8-b (TRPM8 antagonist) instillations significantly reversed the corneal mechanical hyperalgesia and spontaneous eye pain. M8-b also reduced the sustained spontaneous and cold-induced ciliary nerve activity observed in DED mice, as well as inflammation in the cornea and TG (60). In this regard, Kaido et al. further studied the cold perception of the TRPM8 pathway in DEs and found that activating TRPM8 only at the peripheral level was not enough to explain the manifestations of DE-related symptoms of discomfort (70). Higher brain levels may be involved in the occurrence and progression of DE symptoms.

## Chloride Channel-Cftr and DE

CFTR is an important chloride ion and water secretion channel (18, 71). In corneal epithelial cells, CFTR channel activation is important for maintaining the balance of chloride transport and promoting tear secretion (72). It has potential value in the treatment of DEs. The protective effect of CFTR channel-related activators on DEs has been observed in DE animal experiments. Nandoskar et al. found that CFTR was also present in lacrimal gland cells, in which CFTR was significantly expressed in ductal cells, while the expression of CFTR was significantly reduced in an autoimmune dacryoadenitis rabbit model (73). There is an obvious imbalance between the osmotic pressure and chloride ions in the ocular surface of DED. However, the combined administration of CFTR activator (genistein) and vitamin D (calcitriol) can reduce HOS-induced TonEBP (Tonicity—responsive enhancer binding protein), inflammatory gene expression, p-p38, and vitamin D receptor (VDR) degradation in HCECs (74). Furthermore, CFTR activators can significantly improve the ocular surface of mice with DE and even accelerate the repair of damaged corneal epithelium (75). Lee et al. screened a new CFTR activator, isorhamnetin, and reported that it could significantly increase the tear secretion in a mouse DE model, improve the ocular surface injury in mice, and inhibit the expression of IL-1β, IL-8, and TNF-α (76). Flores et al. also screened aminophenyl-1,3,5-triazine, CFTRact-K089, and fully activated CFTR, which can double tear secretion in a DE mouse model (77). In the tear deficiency mouse DE model induced by lacrimal ablation, it is interesting to note that 0.1 nmol CFT act-k089 can restore the tear secretion level by administering drops three times a day (77). Felix et al. conducted pharmacological experiments on New Zealand white rabbits with CFTR activator cftract-k267 and found that CFTR channel activation significantly increased tear production. At the same time, no obvious long-term toxicity affected the ocular surface after continuous treatment for 28 days (78). These data further suggest the relative safety and potential advantages of CFTR-related activators in DE treatment.

## AQPS and DE

AQPs were previously thought to be selective only to water, but recent studies have found that they have more complex regulatory mechanisms and a range of permeability. They also have characteristics of double water channels and gated ion channels and play an important role in maintaining the homeostasis of internal and external balance (21, 79). AQPs are closely related to the transport of Na^+^ and K^+^ on the cell membrane. They can maintain the osmotic gradient of corneal cells, further maintain the water balance of corneal cells, and play an important role in corneal transparency (21, 79). In the corneal epithelium, aquaporins 3 and 5 (AQP3 and 5) have been identified (19, 72, 80–82). Tear film hypertonicity and ocular surface inflammation are the main pathogenic factors of DE, and the AQP5 protein channel plays a role in the pathological process. AQPs and anti-AQPs autoantibodies have been confirmed to be involved in the pathogenesis of Sjögren's syndrome (83). Liu et al. reported that AQP5-/- mice can spontaneously develop DE symptoms, in which AQP5 deficiency changes the structure of lacrimal gland epithelial cells (84). Mucin secreted by conjunctival goblet cells (mainly MUC5AC) is a key condition for tear film stability. The co-immunoprecipitation of conjunctival AQP5 and MUC5AC suggested a possible physical interaction between the two molecules in response to acute DE stress (85). Nakamachi et al. found that pituitary adenylate cyclase-activating polypeptide (PACAP)–deficient mice can have DE-like symptoms, such as corneal keratosis and reduced tears. PACAP eye drops can increase the level of AQP5 in the tear film and p-AQP5 in the infraorbital lacrimal gland. Inhibition of AQP5 can reduce PACAP-induced tear secretion (86). In the study of new DE alternative drugs, it was revealed that the internal mechanism might be related to the upregulation of AQP5. Yu et al. found that ambroxol significantly increased tear secretion and upregulated the expression of AQP5 (87). AQP5 can also be used as a predictor of DE. Mani et al. detected that the expression of AQP4 increased and the expression of AQP5 decreased in conjunctival cells of patients after vitreoretinal surgery, indicating that the changes in these factors may suggest the prognosis of surgically sourced DE (88). Interestingly, Ren et al. found that hyperosmolarity-induced AQP5 upregulation promoted inflammation and caused corneal cell apoptosis (89), which seems to be a contradictory result and may be related to the overall internal imbalance of AQP5.

## Purine and Purinergic Receptors and DE

Purine receptors are widely distributed in neuronal and non-neuronal cells and mediate important signals such as cell proliferation, differentiation, and death, and participate in physiological and pathological activities such as immune response, exocrine and endocrine, inflammation, pain, platelet aggregation, and endothelial-mediated vasodilation (20, 90, 91). Purine receptors mainly include G protein-coupled adenosine receptors (ARS), P2Y receptors (p2yrs), and ATP-gated P2X receptor ion channels (p2xrs).

There are few studies on G protein coupled adenosine receptors (ARS) and P2X receptors in the field of DE (13). A key adaptive response to tear film hyperosmolarity induced by excessive evaporation is the reflective release of mucin by conjunctival goblet cells. The P2X7 receptor/channel is also activated during continuous extracellular hyperosmolarity. The activation of P2X7 not only damages the viability of goblet cells but also enhances exocytosis activity (26).

The P2Y receptor is a purinergic receptor, a G protein coupled receptor of extracellular nucleotides, and participates in physiology and pathophysiology, including inflammatory response and neuropathic pain (92, 93). At present, the P2Y2 subtype in the P2Y receptor (p2yrs) family is closely related to DEs (13). Research has found that the number of purinergic receptors P2Y1, P2Y11, and P2Y13 in lacrimal gland MEC (myoepithelial cells) of TSP1-/- mice (a mouse model of Sjögren's syndrome) decreased significantly, and the regulatory ability of cholinergic agonists, VIP, and purinergic receptors decreased, accompanied by increased expression of inflammatory factors (94). Dogru et al. reported that the tear stability, quantity, and ocular surface health of aged mice decreased with age, but the mRNA expression level of the P2Y2 receptor in the conjunctiva increased significantly, which may be used as compensation for the decline of age-related tear function (95).

Diquafosol is a purinergic P2Y2 receptor agonist that stimulates the conjunctiva to secrete water and mucin. It has achieved positive results in basic and clinical research in the field of DE. The diquafosol tetrasodium (DQS) was found to stimulate meibocytes to release lipids through the P2Y2 receptor and possibly facilitate holocrine secretion in isolated rabbit Meibomian gland cells (96). Diquafosol significantly reduced the levels of reactive oxygen species, apoptosis, and inflammation in corneal cells caused by DE in *in vivo* and *in vitro* DE models (97). In a one-year clinical study, the researchers used diquafosol to treat 580 patients with DEs for 12 months and found that it significantly improved the kerato-conjunctival staining score, tear film break-up time, and Dry Eye-related Quality of Life Score (DEQS) (98). This demonstrated that diquafosol 3.0% ophthalmic solution was tolerated well and was effective in the long-term treatment of DED. Utsunomiya et al. further confirmed that diquafosol was more effective in patients with DEs with foreign body sensation and problems when reading and using visual display terminals (99, 100). In a large randomized, double-blind clinical study, diquafosol improved the ocular surface Rose Bengal staining score more than sodium hyaluronate ophthalmic solution (101).

## Conclusions

**Figure 2:**
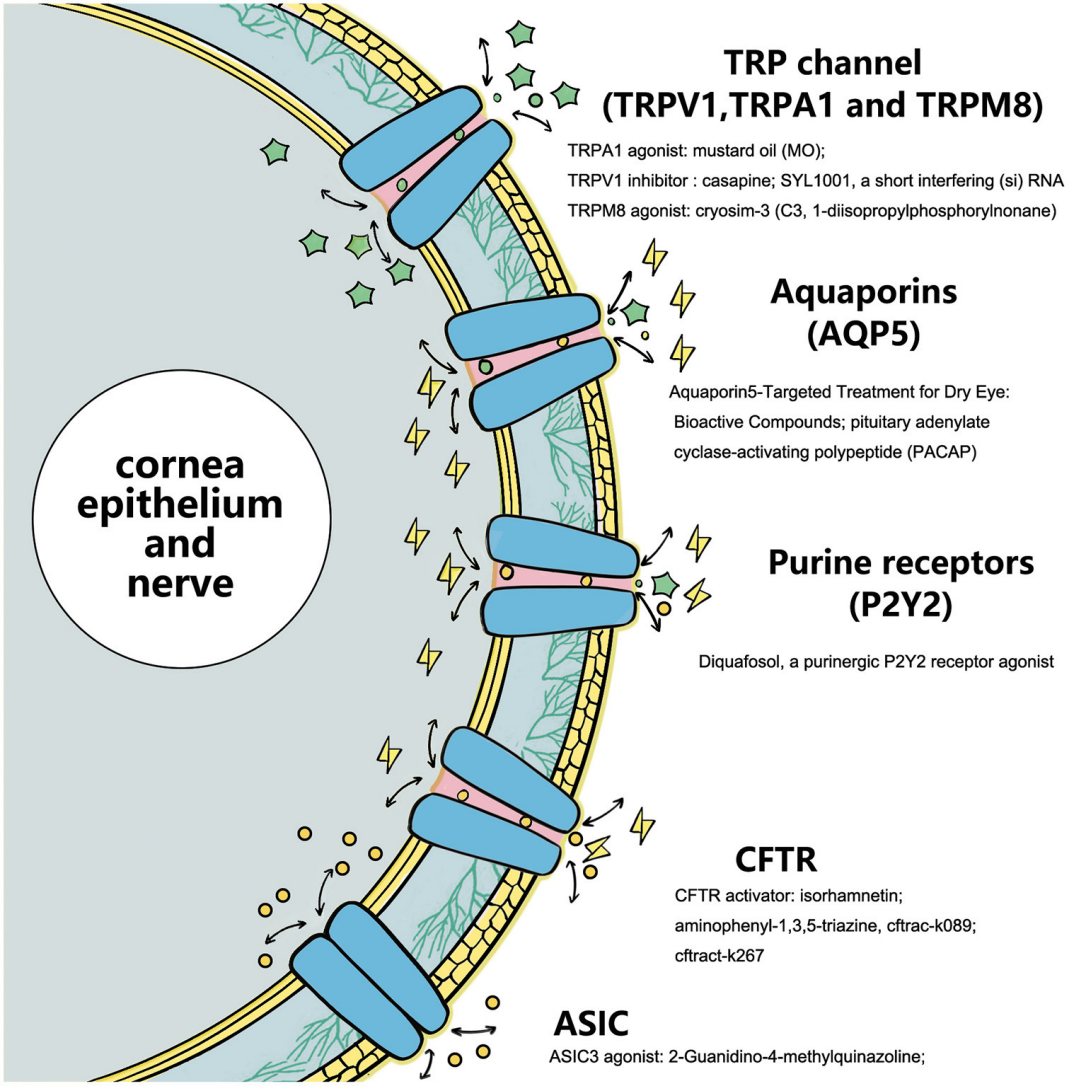
Ion-channels target activating or inhibiting compounds as substrates for these specific receptors affect subsequent ions and intra- and extracellular information transmission.

As an important intercellular information-mediated pathway, ion channels play an important role in various physiological and pathological processes. As the main component of the ocular surface, many ion channels with various functions exist in the corneal nerve, corneal epithelium, and conjunctival epithelium. DE is a common ocular surface disease, and the ion channels play an important role in its pathogenesis. This review summarized the current research status and progress of TRP channels, AQPs, CFTR chloride channels, and purine and purified receptors closely related to DE. Notably, the development of antagonists and activators for these key channels may help with their further popularization and clinical applications. However, from other studies, we have recognized that there may also be possible biological functional variability behind relevant antagonists or activators, which may be related to the overall stability of the internal environment and the interaction between different channel signals. The eye surface receptor protein family has a wide range of members, and its corresponding physiological functions are also diverse. Selection of the best receptor channel, finding the best specific receptor channel targeted drugs, and reducing the impact on other physiological functions will be the focus areas of ion channel research. Therefore, pharmacologists need to screen channel-targeted compounds that are more stable, simple, and have fewer side effects to provide more choices for the over-the-counter treatment of DE. In conclusion, a new and broad treatment protocol for treating DEs by developing ocular surface ion receptor channel-targeted therapy is in our future (Figure 2).

## Author Contributions

SY and XJ conceived the idea and wrote the manuscript. SY, YW, CW, and XJ prepared and reviewed the manuscript. All authors have read and approved the final version of the manuscript.

## Funding

This study was supported by the National Natural Science Foundation of China 81900816 (SY) and the Natural Science Foundation of Zhejiang Province LQ19H120010 (SY).

## Conflict of Interest

The authors declare that the research was conducted in the absence of any commercial or financial relationships that could be construed as a potential conflict of interest.

## Publisher's Note

All claims expressed in this article are solely those of the authors and do not necessarily represent those of their affiliated organizations, or those of the publisher, the editors and the reviewers. Any product that may be evaluated in this article, or claim that may be made by its manufacturer, is not guaranteed or endorsed by the publisher.
